# Impact of obesity severity on postoperative outcomes and recovery progress in patients undergoing unilateral biportal endoscopy for degenerative lumbar disc herniation

**DOI:** 10.3389/fsurg.2025.1598799

**Published:** 2025-05-26

**Authors:** Xiulei Xu, Jun Li, Jie Song, Gang Zhou, Jiren Cai, Xiaorui Zhang

**Affiliations:** Department of Orthopedics, Xinjiang Production and Construction Corps First Division Alar Hospital, Alar City, Xinjiang, China

**Keywords:** degenerative lumbar disc herniation, generalized linear mixed model, obesity, recovery progress, unilateral biportal endoscopy

## Abstract

**Background:**

Obese patients undergoing Unilateral Biportal Endoscopy (UBE) surgery for degenerative lumbar disc herniation may experience postoperative recovery significantly influenced by the degree of obesity and related factors. This study aims to evaluate the impact of obesity severity on postoperative complications and recovery progress following UBE surgery and to identify key intervention points.

**Methods:**

Preoperative baseline characteristics and postoperative follow-up data of patients with mild, moderate, and severe obesity were collected to analyze the incidence of complications, postoperative recovery trajectories, and key influencing factors. Multivariate logistic regression was conducted to examine factors affecting early mobilization (within 24 h), length of hospital stay, and anesthesia recovery time. Generalized linear mixed models (GLMM) were utilized to assess longitudinal changes in postoperative pain, functional disability, walking capacity, and muscle strength over time and their interactions with body mass index (BMI).

**Results:**

Obesity severity was significantly associated with the incidence of postoperative complications. Multivariate logistic regression analysis identified BMI classification, disc calcification, lumbar spondylolisthesis, and inflammatory markers as independent predictors of functional recovery, hospital stay, and anesthesia recovery time. Obese patients showed delayed functional recovery at the 3-month follow-up. Greater obesity severity was associated with slower improvements in walking ability at 1 and 3 months postoperatively. Moreover, obesity severity demonstrated a significant negative correlation with electromyographic activity at 1 month postoperatively.

**Conclusion:**

Obesity severity, inflammation, and anatomical factors are critical determinants of functional recovery in obese patients following UBE surgery. Patients with higher levels of obesity tend to have poorer mid- to long-term outcomes after UBE surgery. For such patients, enhanced postoperative mid- to long-term rehabilitation and physical function recovery are necessary to improve the prognosis of UBE.

## 1 Introduction

Obesity has become an increasingly critical global public health concern, with prevalence rates continuing to rise in recent years (1, 2). According to the World Health Organization (WHO), obesity not only increases the risk of various chronic conditions, such as cardiovascular diseases, diabetes, and metabolic syndrome, but is also closely linked to musculoskeletal disorders (3–5). Moreover, obesity is recognized as a major contributing factor to the development of degenerative spinal diseases (6). Degenerative lumbar disc herniation, one of the most common spinal degenerative conditions, typically presents with persistent back and leg pain, often accompanied by neurological deficits, reduced quality of life, and significant impairment of mobility (7). The pathogenesis of this disease is complex, involving intervertebral disc degeneration, increased mechanical loading, and local inflammatory responses (8). Due to excessive body weight and metabolic dysregulation, obese patients experience a higher incidence of degenerative spinal disease and encounter greater challenges in treatment.

In recent years, unilateral biportal endoscopy (UBE) has emerged as a leading surgical technique for the treatment of degenerative lumbar disc herniation (DLDH) (9, 10). Owing to its minimally invasive nature, enhanced visualization, and reduced soft tissue disruption, UBE has become increasingly favored by spine surgeons (11). Although UBE has demonstrated favorable clinical outcomes in the general population, its efficacy in obese patients may be influenced by a range of factors. Anatomical variations, systemic inflammatory status, postoperative recovery capacity, and an elevated risk of complications in obese individuals may significantly affect surgical prognosis.

Previous studies have investigated the association between obesity and postoperative complications, indicating that obesity is closely linked to an increased incidence of infections, deep vein thrombosis, and chronic postoperative pain. However, most existing research on the efficacy of unilateral biportal endoscopy (UBE) has focused on comparisons with other surgical techniques for spinal disorders, with few studies specifically examining postoperative outcomes and influencing factors in obese patients (12–14). This study aims to address this gap in the literature.

By analyzing postoperative recovery data from patients with mild, moderate, and severe obesity undergoing UBE, this study aims to investigate the impact of obesity severity on postoperative pain, functional impairment, gait recovery, and muscle function. It systematically assesses the association between obesity and postoperative complications, as well as key recovery indicators. Through multivariate regression analysis and generalized linear mixed models (GLMM), the study further identifies critical factors influencing postoperative recovery, including obesity severity, preoperative inflammatory markers (e.g., C-reactive protein, prothrombin time), and anatomical characteristics (e.g., disc calcification and lumbar spondylolisthesis). These analyses seek to elucidate the mechanisms by which obesity affects postoperative recovery, thereby providing scientific evidence to inform individualized postoperative management and rehabilitation strategies in clinical practice.

## 2 Materials and methods

### 2.1 Patient selection

This retrospective study included obese patients who underwent unilateral biportal endoscopy (UBE) surgery for degenerative lumbar disc herniation between January 2020 and January 2023. Patients were randomly categorized into three groups based on body mass index (BMI): Obesity class I (Mild, BMI 30–34.9 kg/m²), Obesity class II (Moderate, BMI 35–39.9 kg/m²), and Obesity class III (Severe, BMI ≥ 40 kg/m²). Inclusion criteria were: age ≥18 years; single-segment lesion; lesion located at L3/L4, L4/L5, or L5/S1; Pfirrmann grade III, IV, or V; and lumbar spondylolisthesis grade ≤ II. Patients were excluded if they had other spinal disorders (e.g., tumors or infections) or severe comorbidities rendering them unfit for surgery.

### 2.2 Data collection

Patient age and gender were collected through the hospital's electronic medical records. Disease duration was calculated based on the patient's chief complaint at the time of outpatient visit or admission. Imaging reports [Magnetic Resonance Imaging (MRI) or Computed Tomography (CT)] were used to determine the Pfirrmann grade, presence of disc calcification, and the degree of lumbar spondylolisthesis. Laboratory indicators, including C-reactive protein (CRP), erythrocyte sedimentation rate (ESR), and prothrombin time (PT), were collected. Postoperative complications such as surgical site infection, deep vein thrombosis, dural tear, nerve injury, chronic postoperative pain, and reoperation rate within one year after surgery were recorded. Pain scores (VAS) and functional disability (ODI) were assessed through questionnaires. The 10-meter walking time was measured using the standard method established by the Rehabilitation Department. Electromyographic peak signals (EMG Peak) were obtained from neurophysiological monitoring reports. Postoperative outcomes including ambulation within 24 h, length of hospital stay, and anesthesia recovery time were also recorded. Pain scores (VAS), functional disability index (ODI), walking time, and EMG Peak values were collected at baseline (preoperative), and at 1 week, 1 month, and 3 months postoperatively. Postoperative one-year ODI and SF-36 scores were collected through questionnaires, and the occurrence of reoperation within one year post-surgery was confirmed via electronic medical records.

Patients with missing BMI data were excluded from the analysis. For missing dynamic outcome variables such as VAS and ODI, multiple imputation was performed under the assumption that the data were missing at random (MAR).

### 2.3 Statistical analysis

Multivariate logistic regression analysis was conducted to identify factors associated with early mobilization (within 24 h postoperatively), length of hospital stay, and anesthesia recovery time. GLMM was used to analyze the dynamic trends of postoperative VAS, ODI, walking time, and EMG Peak over time. BMI group, time points, and other potential confounders were included as fixed effects, while patient ID was set as a random effect. This approach effectively controlled for confounding variables and allowed for a more accurate assessment of the impact of BMI on postoperative recovery.

## 3 Results

### 3.1 Baseline information of patients with different degrees of obesity

The results showed that the incidence of disc calcification was significantly higher in patients with moderate and severe obesity compared to those with mild obesity. The proportion of lumbar spondylolisthesis grades I and II was also significantly higher in the severely obese group than in the mild and moderate obesity groups. Levels of C-reactive protein (CRP), erythrocyte sedimentation rate (ESR), and prothrombin time (PT) varied significantly among patients with mild, moderate, and severe obesity. Other factors, including age, sex, disease duration, and Pfirrmann grade, did not differ significantly among the three groups (Supplementary Table S1).

### 3.2 Differences in postoperative complications among patients with different degrees of obesity

The results indicated that greater obesity severity significantly affected the incidence of postoperative complications. The rates of surgical site infection (SSI) were 1.65%, 7.75%, and 7.25% in the mild, moderate, and severe obesity groups, respectively (*P* = 0.02461). The incidence of deep vein thrombosis (DVT) was 7.25% in severely obese patients, which was significantly higher than in the mild and moderate obesity groups (*P* = 0.00109). Additionally, the incidence of chronic postoperative pain (CPP) increased progressively with obesity severity, rising from 4.4% in mildly obese patients to 21.71% in the severely obese group (*P* = 1.12 × 10^−05^). Similarly, the rate of reoperation within one year postoperatively reached 13.95% in severely obese patients, substantially higher than the 1.65% observed in the mildly obese group (*P* = 1.48 × 10^−04^). These findings indicate that increased obesity severity significantly elevates the risk of surgery-related complications and reoperation (Supplementary Table S2).

### 3.3 Multivariate logistic regression analysis of factors influencing postoperative recovery in obese patients

The results revealed that disc calcification (OR = 0.228, *P* = 0.001), lumbar spondylolisthesis (OR = 0.602, *P* = 0.047), BMI group (OR = 0.189, *P* < 0.001), and PT (OR = 0.579, *P* = 0.011) were significantly associated with a reduced likelihood of mobilization within 24 h postoperatively. Regarding the length of hospital stay, BMI group (OR = 0.586, *P* = 0.001) and CRP levels (OR = 0.871, *P* = 0.025) emerged as key factors contributing to prolonged hospitalization. Anesthesia recovery time was significantly associated with lumbar spondylolisthesis (OR = 0.253, *P* < 0.001), BMI group (OR = 0.533, *P* < 0.001), CRP levels (OR = 0.816, *P* = 0.003), and PT (OR = 0.685, *P* = 0.024), indicating that higher BMI, the presence of lumbar spondylolisthesis, and elevated inflammatory markers contributed to delayed anesthesia recovery. These findings demonstrate that the severity of obesity, inflammatory status, and anatomical factors are critical determinants of postoperative recovery in obese patients, emphasizing the importance of enhanced postoperative management and tailored rehabilitation strategies for high-risk individuals (Supplementary Table S3).

### 3.4 Trend analysis of postoperative recovery indicators in obese patients

**Figure 1:**
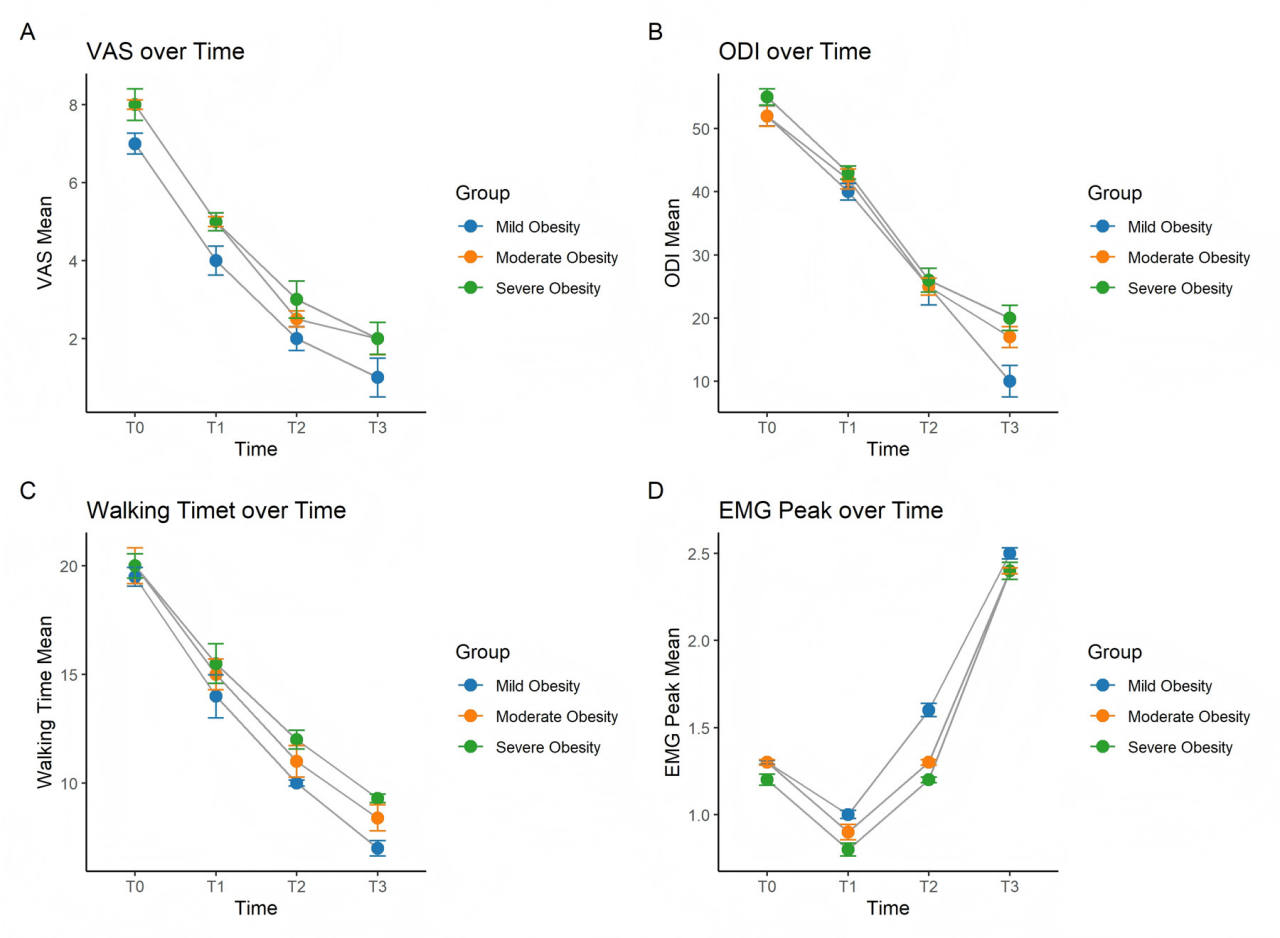
Trends over time in **(A)** VAS, **(B)** ODI, **(C)** walking time, and **(D)** EMG peak among patients with different degrees of obesity.

The results showed that all recovery indicators significantly improved over time (Figures 1A–D). However, functional recovery at 3 months postoperatively was markedly delayed in severely obese patients. Analysis of walking time revealed that greater obesity severity was associated with slower early gait recovery; severely obese patients exhibited significantly longer walking times at both 1 month and 3 months postoperatively compared to the other groups. Electromyographic (EMG) peak signals initially declined after surgery but gradually improved over time, with muscle function recovery in severely obese patients progressing more slowly yet approaching the levels observed in other groups by 3 months. Overall, these findings suggest that higher obesity severity is closely associated with delayed medium- and long-term postoperative recovery.

### 3.5 Generalized linear mixed model analysis of postoperative recovery indicators in obese patients

The results indicated that BMI is a critical factor affecting postoperative recovery. Obese patients exhibited significantly higher VAS scores (*P* = 0.009), worse ODI scores (*P* < 0.001), longer 10-meter walking times (*P* = 0.024), and significantly reduced EMG peak signals (*P* = 0.017). These findings suggest that higher levels of obesity are associated with slower pain relief, poorer functional recovery, delayed gait improvement, and weaker muscle function following surgery. Over time, all recovery indicators improved significantly (*P* < 0.001), demonstrating the time-dependent nature of postoperative recovery. However, analysis of the interaction between BMI and postoperative time revealed that obesity severity was significantly positively correlated with ODI scores at 3 months postoperatively. This indicates that greater obesity severity is associated with more severe functional impairment at 3 months, highlighting the negative impact of obesity on medium- and long-term functional outcomes. For the 10-meter walking time, obesity severity showed a significant positive correlation with walking time at both 1 month and 3 months postoperatively, suggesting that obese patients experience slower gait recovery. The effect was most pronounced at 1 month postoperatively, as evidenced by higher coefficient values compared to those at 3 months, indicating that the impact of obesity on gait recovery is most significant during the early postoperative period. Regarding EMG signals, obesity severity demonstrated a significant negative correlation with EMG peak signals at 1 month postoperatively, suggesting that higher obesity levels are associated with delayed muscle function recovery. However, this association was no longer significant by 3 months postoperatively. In addition to obesity severity, the presence of disc calcification and prolonged prothrombin time (PT) were also significantly negatively correlated with functional recovery, gait recovery, and muscle function recovery (Supplementary Table S4).

### 3.6 Differences in postoperative recovery at one year among patients with different levels of obesity

**Figure 2:**
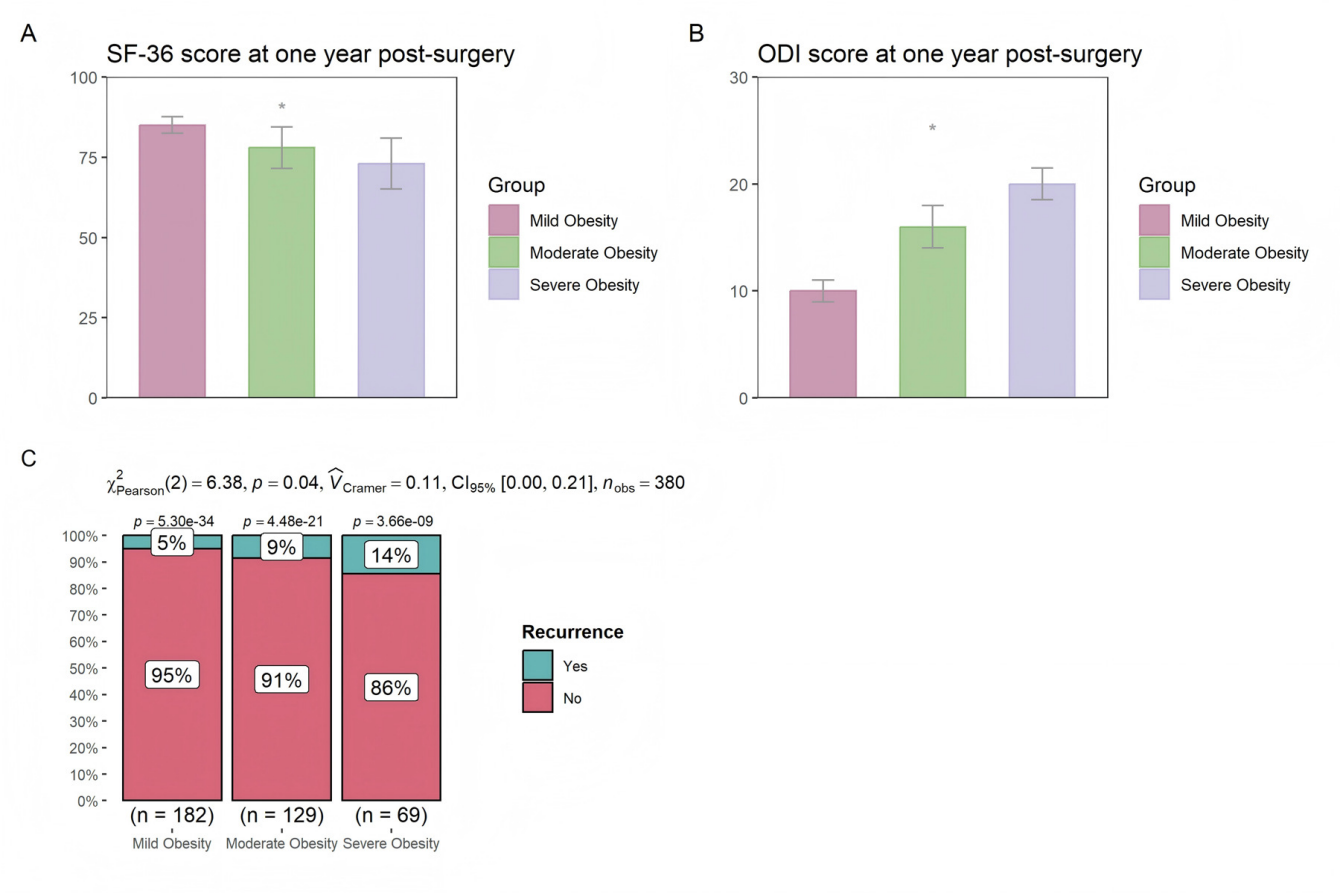
Differences in **(A)** SF-36, **(B)** ODI, and **(C)** reoperation rates at one year post-surgery among patients with different levels of obesity.

The results indicate that the higher the degree of obesity, the lower the quality of life scores at one year post-surgery (Figure 2A); the higher the ODI scores (Figure 2B); and the reoperation rate within one year for patients with severe obesity is 14%, significantly higher than that of those with mild and moderate obesity (Figure 2C). These results suggest that patients with higher levels of obesity have worse long-term outcomes.

## 4 Discussion

This study is the first to systematically analyze the recovery process and influencing factors in obese patients undergoing unilateral biportal endoscopy (UBE) surgery, with a particular focus on the dynamic changes in postoperative recovery indicators, including pain, functional impairment, walking ability, muscle function, and associated complications. The results demonstrated significant delays in postoperative recovery among obese patients, particularly in those with severe obesity, who exhibited marked delays in functional recovery, gait improvement, and muscle function restoration.

The incidence of surgical site infections (SSI), deep vein thrombosis (DVT), and chronic postoperative pain was found to be significantly higher in patients with moderate to severe obesity compared to those with mild obesity. This can be attributed to a combination of factors. Chronic low-grade inflammation weakens immune function, while excessive adipose tissue impairs local blood supply and delays wound healing. Furthermore, prolonged surgical times and increased postoperative care complexity in obese patients may elevate the risk of infections (15, 16). Obesity-associated hypercoagulable states, limited postoperative mobility, and increased venous pressure collectively raise the likelihood of DVT (17, 18). Additionally, the burden of excess weight, insufficient muscle strength, and compensatory abnormal spinal motion patterns contribute to delayed pain relief postoperatively. Chronic inflammation and psychological factors, such as anxiety and depression, further exacerbate the perception of pain, making recovery more challenging for obese patients.

Multivariate logistic regression analysis identified disc calcification, lumbar spondylolisthesis, BMI classification, CRP, and PT as significant factors influencing postoperative recovery, highlighting various underlying mechanisms. Disc calcification and lumbar spondylolisthesis complicate surgical procedures, potentially delaying the efficacy of neural decompression and compromising spinal stability, thereby hindering functional recovery (19, 20). Patients with higher BMI, due to their increased body weight, experience more severe postoperative inflammatory reactions and reduced mobility, further delaying pain relief and gait improvement. Elevated CRP levels indicate chronic inflammation prior to surgery, which may worsen wound healing and prolong pain after the procedure. Prolonged PT reflects preoperative coagulation dysfunction in obese patients, increasing the risk of postoperative bleeding and impairing wound healing, thereby further complicating the recovery process. Collectively, these factors impact postoperative recovery through increased internal pressure, heightened inflammatory responses, and altered metabolic status.

The higher the degree of obesity, the weaker the peak electromyographic (EMG) signals. This may be attributed to the increased release of pro-inflammatory factors such as TNF-α and IL-6 in patients with higher levels of obesity, which inhibit nerve regeneration and lead to slower postoperative recovery of neural signal conduction (21). Additionally, the accumulation of local adipose tissue in obese patients may exert pressure on the lumbar spine. Even after decompression through UBE, chronic compression of the nerve roots may persist postoperatively, resulting in weakened EMG peak signals. Furthermore, lumbar muscle atrophy is more severe in obese LDH patients, requiring a longer time for recovery and further delaying the restoration of EMG peak signals (22).

The interaction analysis indicated that BMI in obese patients had a significant impact on functional disability at 3 months postoperatively, 10-meter walking time at 1 and 3 months, and EMG peak values at 1 month postoperatively. This suggests that the 1-month and 3-month postoperative periods are critical intervention time points (23). At 3 months postoperatively, efforts should focus on lumbar functional rehabilitation; at 1 month, attention should be given to muscle strength and group training; and during both periods, patients should be encouraged to improve daily activity and balance (24). In addition, this finding provides a reference for preoperative preparation and management, helping patients understand the long-term impact of obesity on postoperative recovery. It also supports the recommendation that patients with moderate to severe obesity should ideally reduce their BMI to below 35 before surgery to improve surgical outcomes. Additionally, it provides a scientific foundation for further research into the relationship between obesity and postoperative recovery, as well as postoperative care, fostering interdisciplinary collaboration to optimize management strategies and improve the quality of life for obese patients.

This study holds important significance, as it fills the gap in research regarding postoperative recovery in obese patients undergoing minimally invasive spinal surgery. It clearly demonstrates a negative correlation between the degree of obesity and postoperative functional recovery. This finding provides important reference for the prognostic management and rehabilitation guidance of obese patients receiving UBE surgery. It suggests that clinicians should pay close attention to BMI classification during preoperative evaluations. Furthermore, it clarifies that individualized rehabilitation interventions should be implemented at 1 month and 3 months postoperatively according to the BMI classification of obese patients.

This study is a retrospective analysis and may be subject to selection bias. In addition, the study had a relatively small sample size and did not include non-obese patients as a control group. Moreover, the specific mechanisms underlying delayed recovery in moderately to severely obese patients were not experimentally verified. Future studies should include larger sample sizes and conduct prospective randomized controlled trials as well as mechanistic experiments to further validate the findings of this study.

## 5 Conclusion

The results of this study indicate that patients with moderate to severe obesity experience a higher incidence of postoperative complications following UBE surgery. Key factors influencing postoperative recovery in these patients include disc calcification, lumbar spondylolisthesis, BMI, CRP, and PT. Obesity severity significantly impacts recovery, with delays observed in recovery indicators at both 1 and 3 months postoperatively. These findings provide a scientific foundation for optimizing postoperative management and developing individualized rehabilitation strategies for obese patients.

## Data Availability

The original contributions presented in the study are included in the article/Supplementary Material, further inquiries can be directed to the corresponding author.
