# Supplementary material for: Impact of obesity severity on postoperative outcomes and recovery progress in patients undergoing unilateral biportal endoscopy for degenerative lumbar disc herniation
Source: Front Surg. 2025 May 26;12:1598799. doi: 10.3389/fsurg.2025.1598799 (PMC12146357; doi:10.3389/fsurg.2025.1598799)
Supplement: Supplementary file 1 [file Table1.docx]

| **Table. 1 Baseline information of patients with different degrees of obesity** | | | | | |
| --- | --- | --- | --- | --- | --- |
| **Variable** | **All Patients (n=380)** | **Mild Obesity (n=182)** | **Moderate Obesity (n=129)** | **Severe Obesity (n=69)** | **P-value** |
| **Age** | 49.78 (32.01-71.96) | 48.36 (32.25-71.96) | 49.32 (32.01-70.85) | 53.70 (32.01-71.93) | 0.394 |
| **Gender** |  |  |  |  | 0.0956 |
| Male | 212 (55.79%) | 111 (60.99%) | 69 (53.49%) | 32 (46.38%) |  |
| Female | 168 (44.21%) | 71 (39.01%) | 60 (46.51%) | 37 (53.62%) |  |
| **Hypertension** |  |  |  |  | 0.0614 |
| Yes | 118 (31.05%) | 56 (30.77%) | 33 (25.58%) | 29 (42.03%) |  |
| No | 262 (68.95%) | 126 (69.23%) | 96 (74.42%) | 40 (57.97%) |  |
| **Diabetes** |  |  |  |  | 0.0982 |
| Yes | 145 (38.16%) | 67 (36.81%) | 44 (34.11%) | 34 (49.28%) |  |
| No | 235 (61.84%) | 115 (63.19%) | 85 (65.89%) | 35 (50.72%) |  |
| **Pfirrmann Grading** |  |  |  |  | 0.0669 |
| Grade III | 147 (38.68%) | 71 (39.01%) | 49 (37.98%) | 27 (39.13%) |  |
| Grade IV | 163 (42.89%) | 74 (40.66%) | 65 (50.39%) | 24 (34.78%) |  |
| Grade V | 70 (18.42%) | 37 (20.33%) | 15 (11.63%) | 18 (26.09%) |  |
| **Affected Segment** |  |  |  |  | 0.0923 |
| L3/L4 | 43 (11.32%) | 17 (9.34%) | 21 (16.28%) | 5 (7.25%) |  |
| L4/L5 | 189 (49.74%) | 87 (47.8%) | 68 (52.71%) | 34 (49.28%) |  |
| L5/S1 | 148 (38.95%) | 78 (42.86%) | 40 (31.01%) | 30 (43.48%) |  |
| **History of lumbar spine trauma** |  |  |  |  | 0.1321 |
| Yes | 55 (14.47%) | 23 (12.64%) | 25 (19.38%) | 7 (10.14%) |  |
| No | 325 (85.53%) | 159 (87.36%) | 104 (80.62%) | 62 (89.86%) |  |
| **Course of Disease** |  |  |  |  | 0.1161 |
| <=6 Months | 106 (27.89%) | 52 (28.57%) | 29 (22.48%) | 25 (36.23%) |  |
| >6 Months | 274 (72.11%) | 130 (71.43%) | 100 (77.52%) | 44 (63.77%) |  |
| **Herniation calcification** |  |  |  |  | 0.0448 |
| Yes | 35 (9.21%) | 10 (5.49%) | 15 (11.63%) | 10 (14.49%) |  |
| No | 345 (90.79%) | 172 (94.51%) | 114 (88.37%) | 59 (85.51%) |  |
| **Lumbar Spondylolisthesis** |  |  |  |  | 0.0289 |
| No | 242 (63.68%) | 127 (69.78%) | 82 (63.57%) | 33 (47.83%) |  |
| Grade I | 116 (30.53%) | 45 (24.73%) | 40 (31.01%) | 31 (44.93%) |  |
| Grade II | 22 (5.79%) | 10 (5.49%) | 7 (5.43%) | 5 (7.25%) |  |
| **C-reactive protein (mg/L)** | 6.50 (3.04-9.19) | 6.22 (3.04-9.01) | 6.77 (3.06-9.08) | 7.07 (3.38-9.19) | 0.0404 |
| **Erythrocyte Sedimentation Rate (mm/h)** | 26.41 (15.43-37.18) | 24.72 (15.43-37.18) | 27.11 (15.53-36.96) | 28.82 (15.77-37.12) | 0.0004 |
| **Albumin (g/dL)** | 3.87 (3.20-4.50) | 3.88 (3.21-4.50) | 3.86 (3.20-4.49) | 3.84 (3.22-4.49) | 0.799 |
| **Prothrombin Time** | 12.57 (11.21-13.78) | 12.41 (11.22-13.78) | 12.73 (11.21-13.76) | 12.76 (11.26-13.61) | 0.0329 |
