# Supplementary material for: Impact of obesity severity on postoperative outcomes and recovery progress in patients undergoing unilateral biportal endoscopy for degenerative lumbar disc herniation
Source: Front Surg. 2025 May 26;12:1598799. doi: 10.3389/fsurg.2025.1598799 (PMC12146357; doi:10.3389/fsurg.2025.1598799)
Supplement: Supplementary file 2 [file Table2.docx]

| **Table. 2 The incidence of postoperative complications in patients with different degrees of obesity** | | | | | |
| --- | --- | --- | --- | --- | --- |
| **Complication** | **All Patients (n=380)** | **Mild Obesity (n=182)** | **Moderate Obesity (n=129)** | **Severe Obesity (n=69)** | **P-value** |
| **Surgical Site Infection** |  |  |  |  | 0.02461 |
| Yes | 18 (4.74%) | 3 (1.65%) | 10 (7.75%) | 5 (7.25%) |  |
| No | 362 (95.26%) | 179 (98.35%) | 119 (92.25%) | 64 (92.75%) |  |
| **Deep Vein Thrombosis** |  |  |  |  | 0.00109 |
| Yes | 7 (1.84%) | 1 (0.55%) | 1 (0.78%) | 5 (7.25%) |  |
| No | 373 (98.16%) | 181 (99.45%) | 128 (99.22%) | 64 (92.75%) |  |
| **Dural Tear** |  |  |  |  | 0.00781 |
| Yes | 9 (2.37%) | 1 (0.55%) | 3 (2.33%) | 5 (7.25%) |  |
| No | 371 (97.63%) | 181 (99.45%) | 126 (97.67%) | 64 (92.75%) |  |
| **Nerve Injury** |  |  |  |  | 0.00792 |
| Yes | 6 (1.58%) | 1 (0.55%) | 1 (0.78%) | 4 (5.8%) |  |
| No | 374 (98.42%) | 181 (99.45%) | 128 (99.22%) | 65 (94.2%) |  |
| **Chronic Postoperative Pain** |  |  |  |  | 1.12E-05 |
| Yes | 49 (12.89%) | 8 (4.4%) | 28 (21.71%) | 13 (18.84%) |  |
| No | 331 (87.11%) | 174 (95.6%) | 101 (78.29%) | 56 (81.16%) |  |
| **1-Year Reoperation Status** |  |  |  |  | 1.48E-04 |
| Yes | 27 (7.11%) | 3 (1.65%) | 18 (13.95%) | 6 (8.7%) |  |
| No | 353 (92.89%) | 179 (98.35%) | 111 (86.05%) | 63 (91.3%) |  |
