# Supplementary material for: Impact of obesity severity on postoperative outcomes and recovery progress in patients undergoing unilateral biportal endoscopy for degenerative lumbar disc herniation
Source: Front Surg. 2025 May 26;12:1598799. doi: 10.3389/fsurg.2025.1598799 (PMC12146357; doi:10.3389/fsurg.2025.1598799)
Supplement: Supplementary file 3 [file Table3.docx]

| **Table. 3 Multivariate Logistic Regression Analysis of Factors Influencing Ambulation Within 24 Hours, Length of Stay, and Anesthesia Recovery Time After Surgery^[[1]](#footnote-1)^** | | | | | | | | |
| --- | --- | --- | --- | --- | --- | --- | --- | --- |
| **Clinical indicator** | **Variable** | **estimate** | **Std error** | **statistic** | **P value** | **OR** | **CI-lower** | **CI-upper** |
| **Ambulation Within 24 Hours** | **Herniation calcification** | -1.480 | 0.446 | -3.320 | 0.001 | 0.228 | 0.095 | 0.545 |
|  | **Lumbar Spondylolisthesis** | -0.507 | 0.256 | -1.982 | 0.047 | 0.602 | 0.365 | 0.994 |
|  | **BMI Group** | -1.668 | 0.222 | -7.511 | 0.000 | 0.189 | 0.122 | 0.291 |
|  | **CRP** | -0.141 | 0.087 | -1.622 | 0.105 | 0.869 | 0.732 | 1.030 |
|  | **ESR** | -0.040 | 0.026 | -1.547 | 0.122 | 0.960 | 0.913 | 1.011 |
|  | **PT** | -0.547 | 0.216 | -2.535 | 0.011 | 0.579 | 0.379 | 0.883 |
| **Length of Stay** | **Herniation calcification** | 0.256 | 0.380 | 0.672 | 0.502 | 1.291 | 0.613 | 2.721 |
|  | **Lumbar Spondylolisthesis** | -0.071 | 0.196 | -0.363 | 0.717 | 0.931 | 0.635 | 1.367 |
|  | **BMI Group** | -0.535 | 0.161 | -3.327 | 0.001 | 0.586 | 0.427 | 0.803 |
|  | **CRP** | -0.139 | 0.062 | -2.234 | 0.025 | 0.871 | 0.771 | 0.983 |
|  | **ESR** | -0.024 | 0.018 | -1.288 | 0.198 | 0.977 | 0.942 | 1.012 |
|  | **PT** | -0.076 | 0.157 | -0.489 | 0.625 | 0.926 | 0.682 | 1.259 |
| **Anesthesia Recovery Time** | **Herniation calcification** | -0.124 | 0.419 | -0.297 | 0.767 | 0.883 | 0.388 | 2.008 |
|  | **Lumbar Spondylolisthesis** | -1.376 | 0.211 | -6.515 | 0.000 | 0.253 | 0.167 | 0.382 |
|  | **BMI Group** | -0.630 | 0.163 | -3.876 | 0.000 | 0.533 | 0.387 | 0.732 |
|  | **CRP** | -0.204 | 0.069 | -2.944 | 0.003 | 0.816 | 0.712 | 0.934 |
|  | **ESR** | -0.053 | 0.020 | -2.600 | 0.009 | 0.949 | 0.912 | 0.987 |
|  | **PT** | -0.378 | 0.168 | -2.256 | 0.024 | 0.685 | 0.493 | 0.952 |

1. CRP:C-reactive protein; ESR: Erythrocyte Sedimentation Rate; PT: Prothrombin Time [↑](#footnote-ref-1)
