# Supplementary material for: Impact of obesity severity on postoperative outcomes and recovery progress in patients undergoing unilateral biportal endoscopy for degenerative lumbar disc herniation
Source: Front Surg. 2025 May 26;12:1598799. doi: 10.3389/fsurg.2025.1598799 (PMC12146357; doi:10.3389/fsurg.2025.1598799)
Supplement: Supplementary file 4 [file Table4.docx]

| **Table. 4 Analysis of changes in postoperative recovery indicators in obese patients using a generalized linear mixed model (GLMM)^[[1]](#footnote-1)^** | | | | | | | | | | | | | |
| --- | --- | --- | --- | --- | --- | --- | --- | --- | --- | --- | --- | --- | --- |
| **Clinical indicator** | **output** | **Herniation calcification** | **Lumbar Spondylolisthesis** | **BMI Group** | **CRP** | **ESR** | **PT** | **TimeT1** | **TimeT2** | **TimeT3** | **BMI Group:TimeT1** | **BMI Group:TimeT2** | **BMI Group:TimeT3** |
| **VAS** | **estimate** | 0.021 | 0.003 | 0.514 | 0.006 | 0.000 | 0.002 | -1.002 | -1.401 | -2.799 | 0.011 | 0.057 | -0.005 |
|  | **std error** | 0.037 | 0.018 | 0.028 | 0.006 | 0.002 | 0.014 | 0.041 | 0.041 | 0.041 | 0.039 | 0.039 | 0.040 |
|  | **statistic** | 0.564 | 0.155 | 18.348 | 1.036 | 0.029 | 0.105 | -24.642 | -34.458 | -68.487 | 0.279 | 1.451 | -0.138 |
|  | **P value** | 0.573 | 0.877 | 0.009 | 0.300 | 0.977 | 0.916 | 0.000 | 0.000 | 0.000 | 0.780 | 0.147 | 0.890 |
| **ODI** | **estimate** | 0.004 | 0.000 | 0.608 | 0.005 | -0.001 | 0.008 | -0.569 | -1.029 | -1.971 | 0.017 | 0.006 | 0.042 |
|  | **std error** | 0.016 | 0.007 | 0.012 | 0.003 | 0.001 | 0.006 | 0.017 | 0.017 | 0.017 | 0.017 | 0.017 | 0.017 |
|  | **statistic** | 0.228 | -0.016 | 50.697 | 1.860 | -0.796 | 1.320 | -32.961 | -59.586 | -114.089 | 1.027 | 0.352 | 2.488 |
|  | **P value** | 0.008 | 0.987 | 0.000 | 0.063 | 0.426 | 0.187 | 0.000 | 0.000 | 0.000 | 0.304 | 0.725 | 0.013 |
| **Walking Time** | **estimate** | 0.002 | 0.001 | 0.005 | 0.000 | 0.000 | 0.003 | -0.561 | -0.886 | -1.108 | 0.004 | 0.037 | 0.003 |
|  | **std error** | 0.003 | 0.001 | 0.002 | 0.000 | 0.000 | 0.001 | 0.003 | 0.003 | 0.003 | 0.003 | 0.003 | 0.003 |
|  | **statistic** | 0.621 | 0.526 | 2.263 | 0.517 | -0.615 | 2.340 | -182.836 | -288.461 | -360.842 | 1.429 | 12.417 | 1.093 |
|  | **P value** | 0.535 | 0.599 | 0.024 | 0.605 | 0.539 | 0.019 | 0.000 | 0.000 | 0.000 | 0.153 | 0.009 | 0.027 |
| **EMG** | **estimate** | -0.005 | -0.001 | -0.005 | -0.001 | 0.000 | -0.004 | -0.401 | 0.259 | 0.558 | -0.001 | -0.017 | -0.005 |
|  | **std error** | 0.005 | 0.002 | 0.004 | 0.001 | 0.000 | 0.002 | 0.005 | 0.005 | 0.005 | 0.005 | 0.005 | 0.005 |
|  | **statistic** | -0.919 | -0.218 | -1.383 | -1.598 | -1.665 | -2.159 | -74.119 | 47.911 | 103.076 | -0.222 | -3.309 | -0.984 |
|  | **P value** | 0.036 | 0.828 | 0.017 | 0.110 | 0.096 | 0.031 | 0.000 | 0.000 | 0.000 | 0.824 | 0.019 | 0.325 |

1. CRP:C-reactive protein; ESR: Erythrocyte Sedimentation Rate; PT: Prothrombin Time; VAS: Pain Visual Analog Scale; ODI: Oswestry Disability Index; EMG: Electromyography [↑](#footnote-ref-1)
